# Evaluation of the bioconversion of genetically modified switchgrass using simultaneous saccharification and fermentation and a consolidated bioprocessing approach

**DOI:** 10.1186/1754-6834-5-81

**Published:** 2012-11-12

**Authors:** Kelsey L Yee, Miguel Rodriguez Jr, Timothy J Tschaplinski, Nancy L Engle, Madhavi Z Martin, Chunxiang Fu, Zeng-Yu Wang, Scott D Hamilton-Brehm, Jonathan R Mielenz

**Affiliations:** 1 Biosciences Division, Oak Ridge National Laboratory, Oak Ridge, TN, 37831-6226, USA; 2 BioEnergy Science Center, Oak Ridge National Laboratory, Oak Ridge, TN, 37831-6226, USA; 3 Forage Improvement Division, The Samuel Roberts Noble Foundation, Ardmore, OK, 73401, USA

**Keywords:** Transgenic, Switchgrass, Fermentation, Consolidated bioprocessing, *Saccharomyces cerevisiae*, *Clostridium thermocellum*, *Caldicellulosiruptor obsidiansis*, *Caldicellulosiruptor bescii*

## Abstract

**Background:**

The inherent recalcitrance of lignocellulosic biomass is one of the major economic hurdles for the production of fuels and chemicals from biomass. Additionally, lignin is recognized as having a negative impact on enzymatic hydrolysis of biomass, and as a result much interest has been placed on modifying the lignin pathway to improve bioconversion of lignocellulosic feedstocks.

**Results:**

Down-regulation of the caffeic acid 3-*O*-methyltransferase (COMT) gene in the lignin pathway yielded switchgrass (*Panicum virgatum*) that was more susceptible to bioconversion after dilute acid pretreatment. Here we examined the response of these plant lines to milder pretreatment conditions with yeast-based simultaneous saccharification and fermentation and a consolidated bioprocessing approach using *Clostridium thermocellum*, *Caldicellulosiruptor bescii* and *Caldicellulosiruptor obsidiansis*. Unlike the *S. cerevisiae* SSF conversions, fermentations of pretreated transgenic switchgrass with *C. thermocellum* showed an apparent inhibition of fermentation not observed in the wild-type switchgrass. This inhibition can be eliminated by hot water extraction of the pretreated biomass, which resulted in superior conversion yield with transgenic versus wild-type switchgrass for *C. thermocellum*, exceeding the yeast-based SSF yield. Further fermentation evaluation of the transgenic switchgrass indicated differential inhibition for the *Caldicellulosiruptor* sp. strains, which could not be rectified by additional processing conditions. Gas chromatography–mass spectrometry (GC-MS) metabolite profiling was used to examine the fermentation broth to elucidate the relative abundance of lignin derived aromatic compounds. The types and abundance of fermentation-derived-lignin constituents varied between *C. thermocellum* and each of the *Caldicellulosiruptor* sp. strains.

**Conclusions:**

The down-regulation of the COMT gene improves the bioconversion of switchgrass relative to the wild-type regardless of the pretreatment condition or fermentation microorganism. However, bacterial fermentations demonstrated strain-dependent sensitivity to the COMT transgenic biomass, likely due to additional soluble lignin pathway-derived constituents resulting from the COMT gene disruption. Removal of these inhibitory constituents permitted completion of fermentation by *C. thermocellum*, but not by the *Caldicellulosiruptor* sp. strains. The reason for this difference in performance is currently unknown.

## Background

Lignocellulosic biomass is an abundant, low-cost, and renewable source of carbon that, when converted into biofuels and biomaterials, has the potential to replace petroleum-based energy sources and materials [1-4]. The high degree of recalcitrance remains a major hurdle to a cost-effective microbial bioconversion of lignocellulosic feedstocks. Lignin is a major component of plant cell walls and impedes enzymatic hydrolysis of the cellulose and hemicellulose to fermentable sugars. There is an inverse relationship between lignin content/composition and plant cell wall enzymatic hydrolysis and fermentation kinetics [5,6]. The evaluation of *Miscanthus sinensis* and *Populus* sp. with varying lignin content and/or alteration of lignin composition showed that sugar release increased as lignin content decreased [7-9]. Similarly, the evaluation of transgenic lines of alfalfa down-regulated in the lignin pathway has shown increased sugar release from hydrolysis in comparison to the wild-type, and this phenomenon is directly related to the reduction of lignin content [10]. A C3′H deficient REF8 mutant of *Arabidopsis* sp. displayed increased susceptibility of enzymatic hydrolysis in comparison to the wild-type [11]. Moreover, the reduction of ferulate-lignin cross-linking or lignin content improved ruminal fermentation performance [6]. Finally, a transgenic switchgrass (*Panicum virgatum*) with down-regulation of the COMT (caffeic acid 3-*O*-methyltransferase) gene showed improved susceptibility to bioconversion using yeast-based simultaneous saccharification and fermentation (SSF) and consolidated bioprocessing (CBP) with *C. thermocellum*[12].

Even though improvements have been made to reduce the cost of hydrolytic enzymes, a CBP approach could mitigate the need for the addition of exogenous hydrolytic enzymes and further reduce biofuel production costs [13-15]. *Clostridium thermocellum*, *Caldicellulosiruptor obsidiansis* and *Caldicellulosiruptor bescii* are thermophilic and cellulolytic gram-positive bacteria. They are CBP candidates because of their ability to ferment biomass substrates without the addition of exogenous enzymes. However, their main fermentation products are a mixture of organic acids (primarily acetic and lactic acid) and ethanol with different product ratios depending on the specific microorganism. These microorganisms require further strain development to become industrially relevant. Characterization of growth and examination of the cellulolytic systems on different substrates for *C. bescii* and *C. obsidiansis* have shown that both microorganisms utilize hexose and pentose sugars, grow on crystalline cellulose, and ferment biomass substrates [16-21]. Examination of the fermentation performance of *C. thermocellum* on cellobiose or crystalline cellulose showed rapid substrate utilization, and in addition, *C. thermocellum* has been shown to utilize up to 75% of the cellulose contained in pretreated biomass substrates [12,15,19,22,23].

In this study, we expanded the fermentation work of Fu et al. [12] to include different cellulolytic bacteria, and a less severe hot water pretreatment, which will likely reduce acid-derived, potentially inhibitory byproducts. Three switchgrass lines with different levels of COMT down-regulation were examined using conventional yeast-based SSF and a CBP approach with *C. thermocellum*, *C. bescii*, and *C. obsidiansis.* We observed considerably different fermentation capabilities of these diverse microorganisms when using native and transgenic switchgrass as substrates.

## Results

Down-regulation of the COMT gene in switchgrass decreased the lignin content, reduced the S/G ratio, increased sugar release, and improved the bioconversion yield after dilute acid pretreatment for yeast-based SSF on the switchgrass lines T1-2, 3, and 12 and CBP with *C. thermocellum* on switchgrass line T1-3 [12]. In this study, two highly down-regulated lines (T1-2 and T1-3) and a moderately down regulated line (T1-12) were evaluated for susceptibility to microbial bioconversion. This was accomplished using two different types of pretreatment conditions, dilute acid (DA) and hot water (HW), and two different fermentation strategies: conventional yeast-based SSF and a CBP approach with *C. thermocellum*, *C. bescii*, and *C. obsidiansis*.

### Simultaneous saccharification and fermentation

**Figure 1:**
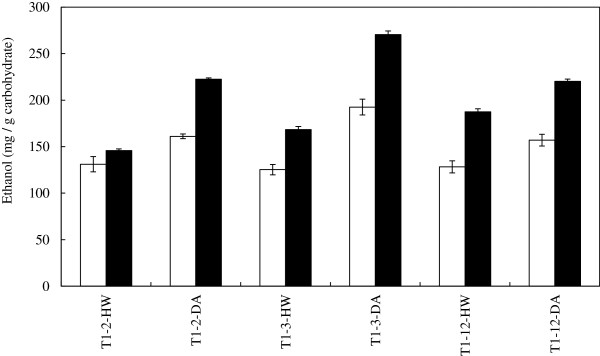
**The effect of pretreatment conditions on the yield of ethanol *S. cerevisiae *yeast-based SSF of wild-type and transgenic switchgrass lines T1-2, T1-3, and T1-12; COMT transgenic (TG) in black bar; wild-type (WT) in white bar; dilute acid (DA); hot water (HW).**

Transgenic (TG) and wild-type (WT) control switchgrass lines were DA pretreated and washed solids were subjected to SSF. The biological triplicate fermentations were monitored by measuring weight loss over time (data not shown). The SSF of transgenic lines had a faster fermentation rate and greater ethanol yield (mg/g cellulose) than their respective control lines of 53%, 61%, and 18% (Figure 1 and Additional file 1: Table S1).

To further investigate the increase in bioconversion susceptibility of the transgenic switchgrass and evaluate the use of a milder pretreatment strategy, the switchgrass lines were HW pretreated and washed. The resulting solids were evaluated by SSF and the transgenic lines T1-2, T1-3, and T1-12 produced more ethanol and had a yield increase of 19%, 54%, and 22%, respectively over their control lines (Figure 1 and Additional file 1: Table S2). The weight loss time course profile for HW pretreated substrates had a similar pattern compared to the DA pretreated biomass with the transgenic lines outperforming their respective controls (weight loss data not shown), although the magnitude of the weight loss for HW was less than that of DA pretreated materials. Therefore, the pretreatment did not impact the COMT effect. However, the severity of pretreatment did impact the final yield, and as a result, the percentage of theoretical yield achieved was greater for SSF of DA in comparison to HW pretreated switchgrass (Figure 1 and Additional file 1: Table S1 and S2).

### Consolidated bioprocessing

Consolidated bioprocessing is considered a lower cost process for biomass fermentation due to fewer unit operations and little or no exogenous enzyme addition [13,24]. A CBP approach was used to evaluate the COMT transgenic switchgrass lines using the thermophilic, anaerobic and cellulolytic microorganisms, *C. thermocellum*, *C. bescii*, and *C. obsidiansis*. For the following CBP platform fermentations describe in this work, no exogenous enzyme was added, and the fermentations were performed in biological triplicate. The fermentation products for the three microorganisms were acetic acid, lactic acid, and ethanol. The ratio of these products varies by microorganism and is shown in Additional file 1: Table S1 and S2. As a result, the yields were reported as a sum of the fermentation products for comparison of the digestibility of the substrate.

**Figure 2:**
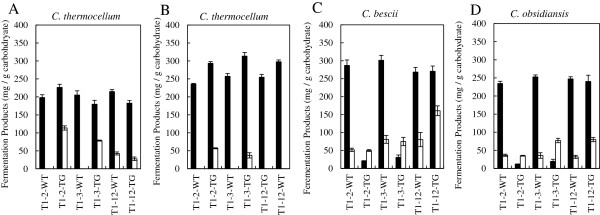
**Comparison of fermentation products yield for CBP conversion of dilute acid pretreated T1-2, T1-3, and T1-2 wild-type (WT) and transgenic (TG) switchgrass with *C. thermocellum*, *C. bescii*, and *C. obsidiansis*.** (**A**) Final total products yield for *C. thermocellum*. (**B**) Final total products yield for *C. thermocellum* with hot water extraction of biomass. (**C**) Final total products yield for *C. bescii* with hot water extraction of biomass. (**D**) Final total products yield for *C. obsidiansis* with hot water extraction of biomass. The black bar represents yield of total fermentation products acetic acid, lactic acid, and ethanol and the white bar represents total residual sugars; glucose plus cellobiose for *C thermocellum*; all biomass sugars for *Caldicellulosiruptor sp* strains.

The same batch of DA pretreated switchgrass used for yeast-based SSF experiments was utilized for fermentations with *C. thermocellum*. The wild-type switchgrass lines yielded 200–225 mg fermentation products/g carbohydrate (Figure 2A and Additional file 1: Table S1). From previous SSF experiments, it was expected that the fermentation of transgenic lines would have an increase in yield over their respective control. However, the fermentation of T1-2, T1-3 and T1-12 transgenics produced yield differences of +14%, –13%, and −15%, respectively, in comparison to their control (Figure 2A). Analysis of the fermentation broths from the highly down regulated T1-2 and T1-3 lines detected significant levels of unfermented glucose and cellobiose although the weight loss data showed the fermentation had ceased. These unfermented carbohydrates likely account for the yield reduction seen in these fermentations. By comparison, both the transgenic and wild-type switchgrass T1-12 lines showed lower residual liberated, but unconsumed sugars (Figure 2A).

**Figure 3:**
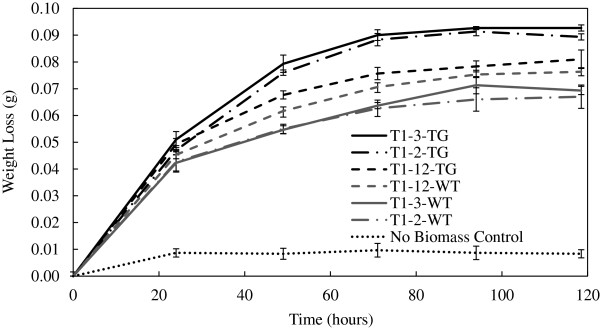
**Fermentation weight loss over time of *C. thermocellum *growing on dilute acid pretreated and hot water extracted T1-2, T1-3, and T1-12 wild-type (WT) and transgenic (TG) switchgrass.**

The nature of the reduced fermentation performance was further examined by attempting to remove possible water soluble inhibitory compounds remaining after pretreatment and initial washing by using hot water extraction. The additional hot water extraction step improved the *C. thermocellum* fermentation of all transgenic lines compared to their respective wild-type lines with the transgenic T1-2, T1-3, and T1-12 producing 25%, 22%, and 18% more total products, respectively (Figure 2B). Furthermore, the T1-2 and 3 transgenic substrates showed a reduced level of residual free sugars compared to the results without hot water extraction. Examination of the weight loss data during fermentations showed all the transgenic substrates fermented more quickly than wild-type substrates and had a larger final weight loss than their respective control implying that the transgenic switchgrass was more susceptible to bioconversion (Figure 3). These results show the additional hot water extraction apparently removed the majority of, heretofore, unidentified inhibitors and improved fermentation performance. Interestingly, if the liberated free sugars were consumed (based upon only glucose conversion to fermentation products), the yield in mg total product/g carbohydrate for *C. thermocellum* fermentations without hot water extraction would have increased, but still less than the yield for fermentations with hot water extraction. This implies that the extent of hydrolysis, as well as the yield, were impacted by these extracted (inhibitory) compounds (Figure 2A and 2B).

There was an improved susceptibility for bioconversion of the transgenic switchgrass over the control for fermentations with *S. cerevisiae* and *C. thermocellum*, which are strictly hexose sugar utilizers. This led to the characterization of fermentation performance of the switchgrass by the *Caldicellulosiruptor* sp. strains, because unlike *C. thermocellum* and *S cerevisiae*, they utilize both hexose and pentose sugars. In addition, they have a significantly higher fermentation temperature optimum (78°C) and a different hydrolytic system than *C. thermocellum*[16-23].

The same switchgrass sources processed identically with DA pretreatment, HW extraction, and extensive washing were subjected to fermentation with *C. obsidiansis* and *C. bescii*. The fermentation of the wild-type switchgrass lines by both *C. bescii* and *C. obsidiansis* yielded approximately 200–225 mg fermentation products/g carbohydrate with minimal residual sugars in the fermentation broth (Figure 2C and 2D and Additional file 1: Table S1). By comparison, fermentation of the highly down-regulated transgenic lines, T1-2 and T1-3, by these *Caldicellulosiruptor* sp. strains had minimal weight loss, indicating reduced fermentation performance (data not shown), that produced less than 50 mg total products/g carbohydrate. In addition, significant levels of unfermented free sugars were detected in the fermentation broth (Figure 2C and Figure 2D). Also, the moderately down-regulated COMT transgenic line T1-12 did not show an improved yield over the control, and had a higher concentration of residual liberated sugar, especially in *C. bescii* fermentations (Figure 2C and 2D). Since the T1-2 and 3 transgenic lines showed both low levels of liberated, but unfermented free sugar, as well as low product yields, it appears that both hydrolysis and fermentation are negatively impacted in comparison to the wild-type line.

**Figure 4:**
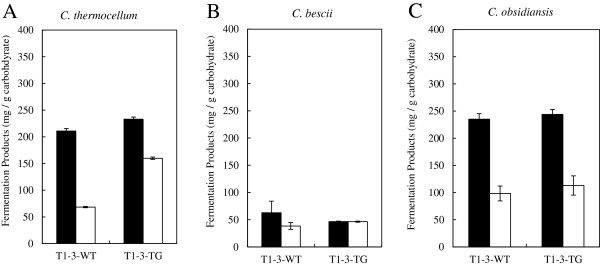
**Comparison of fermentation products yield for CBP conversion of hot water pretreated, hot water extracted T1-3 wild-type (WT) and transgenic (TG) switchgrass with *C. thermocellum *(A), *C. bescii *(B), and *C. obsidiansis *(C).** The black bar represents yield of total fermentation products acetic acid, lactic acid, and ethanol and the white bar represents total residual sugars; glucose plus cellobiose for *C thermocellum*; all biomass sugars for *Caldicellulosiruptor sp* strains.

It was clear that the three CBP candidate microorganisms were inhibited to varying levels during bioconversion of the DA, HW extracted and extensively washed transgenic switchgrass solids, which was not observed in yeast-based SSF. As a result, fermentations with a less severe hot water pretreated T1-3 feedstock line (T1-3-WT and T1-3-TG) with the three bacteria were performed to examine if a less severe pretreatment minimized the fermentation inhibition patterns observed with DA pretreated switchgrass. Using the identical batch of pretreated substrates tested with yeast-based SSF, fermentations with all three aforementioned CPB bacteria were completed. The fermentation of the wild-type and transgenic line by *C. thermocellum* showed the transgenic line produced 10% more total fermentation products/g carbohydrate than the control (Figure 4A and Additional file 1: Table S2). The weight loss was monitored over time and showed the fermentation of the transgenic lines had marginally faster rates and greater total weight loss, further supporting that the fermentation performance was slightly better than the wild-type line (data not shown). However, we detected significant levels of liberated, but unfermented sugars in the fermentation broths from the wild-type and transgenic feedstocks. There was significantly higher concentration of residual sugars in the transgenic fermentation broth implying that the material was more susceptible to hydrolysis, but apparently had a higher degree of inhibition of sugar fermentation. The theoretical yield for the fermentation of the transgenic switchgrass, if all the residual glucose was utilized, would have been 313 mg total products/g carbohydrate or a 28% increase in comparison to the control line at 245 mg total products/g carbohydrate. Therefore, the fermentation yield from the HW pretreated transgenic line is comparable to that from the DA pretreated line, which had a yield of 332 mg total products/g carbohydrate.

The same HW pretreated and washed biomass source used in the previous fermentations was evaluated for bioconversion susceptibility with the *Caldicellulosiruptor* sp. strains. The fermentation of the transgenic and wild-type line with *C. bescii* again displayed low fermentation yields of approximately 50 mg total product/g carbohydrate (Figure 4B and Additional file 1: Table S2). In addition, as with the DA pretreatment, there was minimal residual unfermented sugar, indicating that both the hydrolysis and fermentation were negatively impacted. However, the *C. obsidiansis* fermentation performance was improved for both the transgenic and wild-type feedstocks yielding approximately 225 mg total product/g carbohydrate with the transgenic biomass providing a 4% greater yield (Figure 4C and Additional File 1: Table S2). Interestingly, there were approximately equal levels of residual sugar in the broths from the fermentation of the transgenic and wild-type feedstocks, so *C. obsidiansis* did not show an increase in bioconversion susceptibility of the transgenic feedstock. Therefore, the *Caldicellulosiruptor sp*. strains showed a different fermentation pattern with HW pretreated biomass compared to the DA pretreated biomass.

### Gas chromatography–mass spectrometry (GC-MS) analysis

There was a differential of fermentation inhibition between the bacteria for both DA and HW pretreated switchgrass shown either by incomplete fermentation of residual free sugars or failure to both hydrolyze and ferment the biomass. This inhibitory behavior was not detected in yeast-based SSF. The reduced fermentation and/or hydrolysis performance was an unexpected result and may have several contributing factors.

Previously, a novel monolignol analog, *iso*-sinapyl alcohol and related metabolites were detected by GC-MS and found to accumulate in the transgenic switchgrass, due to the block in the lignin biosynthetic pathway, and had mild-inhibitory properties towards yeast and *E. coli*[25]. In order to gain insight into this and other possible bacterial inhibitors, GC-MS-based metabolite profiling was conducted to analyze the biochemical constituents in the fermentation broth. End point fermentation samples were analyzed after fermentations with all three CBP candidate microorganisms using both DA and HW pretreated transgenic and control switchgrass substrates. The newly discovered monolignol analog (*iso*-sinapyl alcohol) was not detected in the fermentation broths from the extensively washed pretreated biomass likely due to its successful extraction. However, there were a large number of aromatic lignin-derived inhibitory constituents in each sample. We have included in our analysis only metabolites that can be identified, are statistically significant (p-value < 0.05), and show at least a 2-fold comparative difference for the microbe-to-microbe analysis on a single switchgrass line (microbe effect) or COMT transgenic versus wild-type switchgrass with a single microorganism (COMT biomass effect).

Prior to analyzing the chemical constituents of the CBP fermentation samples, appropriate parallel triplicate controls were analyzed. Positive (biomass and no cells) and negative (no biomass and cells) controls in media at the three different fermentation temperatures (35°C, 58°C, and 75°C) were analyzed and the GC-MS data showed media components and minimal quantities of a few carbohydrates for the positive controls. In addition, parallel triplicate controls containing biomass treated with fungal hydrolytic enzymes were analyzed and showed only media components and carbohydrates (data not shown).

The metabolite profiles for the fermentation of HW pretreated switchgrass lines indicated at least seven possible inhibitory aromatic or mono-phenolic compounds. The effect of the COMT down-regulation (biomass effect) was evaluated by calculating the ratio of the constituent in the transgenic switchgrass to the wild-type for each microorganism. The ratio for the biomass effect of the constituents for the identifiable compounds did not show differentials that were statistically significant with ratios greater than 2-fold except for a C5 sugar-sinapyl-conjugate from the *C. obsidiansis* fermentation (Additional file 2: Table S3). The evaluation of the biomass effect showed approximately equivalent relative abundance of aromatic constituents in the fermentation of transgenic and wild-type lines for a single microbe. This is consistent with the fermentation yields not being as large as 2-fold difference between the transgenic and control lines. However, this does not explain the differential of fermentation inhibition between the CBP candidate microorganisms.

In order to further evaluate the apparent inhibition, the microbe effect was evaluated by comparing the ratio of aromatic compounds detected in each switchgrass line for each microorganism (Table 1, Additional file 2: Table S4 and S5). The ratio of the *Caldicellulosiruptor* sp. strains to *C. thermocellum* by feedstock line showed several identifiable compounds (C5-sugar-sinapyl-conjugate, 5-hydroxyconiferyl alcohol, and coniferyl alcohol) and many unidentified constituents that have greater than a 2-fold statistically significant increase. Overall, the switchgrass fermentations by *Caldicellulosiruptor* sp. strains are liberating a larger relative abundance of likely inhibitory aromatic conjugates and mono-phenolic acid constituents as they hydrolyze the biomass in comparison to *C. thermocellum*. This may partially account for the reduced fermentation performance of *C. bescii* and the lack of COMT effect seen in the fermentations with *C. obsidiansis*. In comparing the microbe effect between *C. bescii* and *C. obsidiansis* (Additional file 2: Table S5), *C. obsidiansis* had a significant increase in arabitol and an arabitol phenolic conjugate, whereas *C. bescii* had a significant increase in C5-sugar-sinapyl conjugate, but no other large change in aromatic constituents to account for the differential fermentation performance between the two *Caldicellulosiruptor sp*. strains.

**Table 1:** **Ratio of selected lignin constituents with a 2-fold comparable difference and p-value < 0.05 after fermentation of hot water pretreated T1-3 switchgrass by*C. bescii*or*C. obsidiansis*versus*C. thermocellum*(microbe effect); transgenic (TG); wild-type (WT) switchgrass**

**Aromatic Constituent (μg / mL) sorbitol equivalents**	***C. bescii*(TG) /*C. thermocellum*(TG)**		***C. obsidiansis*(TG) /*C. thermocellum*(TG)**		***C. bescii*(WT) /*C. thermocellum*(WT)**		***C. obsidiansis*(WT) /*C. thermocellum*(WT)**	
	**Ratio**	**P-value**	**Ratio**	**P-value**	**Ratio**	**P-value**	**Ratio**	**P-value**
arabitol	0.11	0.00	1.27	0.28	0.086	0.00	1.19	0.00
p-coumaric acid	0.43	0.00	0.31	0.00	0.26	0.01	0.19	0.00
C5-sugar-sinapyl conjugate	67.17	0.00	8.58	0.00	72.34	0.01	6.61	0.00
5-hydroxyconiferyl alcohol	17.01	0.00	19.66	0.00	16.66	0.00	11.92	0.00
coniferyl alcohol	3.68	0.00	2.49	0.00	3.44	0.00	1.97	0.00

The metabolite profiles for fermentation samples of DA pretreated feedstocks showed eight identifiable aromatic conjugates or mono-phenolic acids that are likely inhibitory compounds. The fermentation samples from HW pretreated switchgrass had only three common identifiable compounds, arabitol, *p*-coumaric acid, and sinapyl alcohol. In evaluating the biomass effect, there was not a strong trend among identifiable compounds from the transgenic versus the wild-type fermentations (Additional file 2: Table S6). However, there was a tentatively identified compound, coumaroyl-benzaldehyde that was two-fold higher in the transgenic versus the wild-type fermentations for all three microorganisms (Table 2). An increase in this aromatic constituent in the transgenic versus the wild-type does not necessarily explain the reduction in fermentation performance shown in the transgenic T1-2 and T1-3 fermentations in comparison to the wild-type lines for the *Caldicellulosiruptor* sp. strains. Moreover, in contrast to the HW pretreated feedstock samples, there is no indication of a notable trend in increase of mono-phenolics and aromatic constituents in the *Caldicellulosiruptor* sp. fermentations versus *C. thermocellum* or the *Caldicellulosiruptor* sp. strains versus each other when fermentations were conducted with DA pretreated feedstocks (Additional file 2: Table S7 and S8). Interestingly, coumaroyl-benzaldehyde was not identified in the fermentation of HW pretreated feedstocks, but was present in greater levels in the DA pretreated feedstock fermentations by the *Caldicellulosiruptor* sp. strains versus *C. thermocellum*, and also increased when the biomass effect was examined (transgenic versus wild-type). Finally, of particular interest was the presence of arabitol, which can be inhibitory, in all bacterial fermentation samples regardless of pretreatment conditions and microorganism. The three microorganisms likely produced arabitol from arabinose.

**Table 2:** Ratio of selected lignin constituents with a 2-fold comparable difference and p-value < 0.05 from transgenic (TG) versus wild-type (WT) T1-3 switchgrass fermentation after dilute acid pretreatment for a specified microbe (biomass effect)

**Aromatic Constituent (μg / mL) sorbitol equivalents**	***C. bescii*(COMT3 TG/WT)**		***C. obsidiansis*(COMT3 TG/WT)**		***C. thermocellum*(COMT3 TG/WT)**	
	**Ratio**	**P-value**	**Ratio**	**P-value**	**Ratio**	**P-value**
coumaroyl-benzaldehyde	2.54	0.01	3.71	0.00	NA	0.00

## Discussion

The combination of a feedstock with increased enzymatic digestibility in combination with the CBP approach, which will eliminate the need for exogenous hydrolytic enzymes, has the potential to further reduce the cost of biofuels. Therefore we examined the fermentation performance of both wild-type and transgenic switchgrass lines using *Clostridium thermocellum*, *Caldicellulosiruptor obsidiansis*, and *Caldicellulosiruptor bescii.* Using three lines of switchgrass down-regulated in the COMT gene [12], we have shown that a milder pretreatment process does not impact the improved product yield generated by fermentation of the COMT down-regulated switchgrass biomass during yeast-based SSF. However, when a CBP-capable bacterium is tested, a significant differential of fermentation inhibition is detected, as judged by product yield on carbohydrate. In the case of *C. thermocellum* fermentations of dilute acid pretreated feedstock, the cellulosome and/or free carbohydrolases appear functional, as indicated by high levels of liberated unfermented glucose and cellobiose in the fermentation broth. At the same time, COMT transgenic feedstock lines clearly generate greater inhibition compared to the wild-type switchgrass, in the case of *C. thermocellum* fermentation. The inhibition of fermentation was shown to be removed after hot water extraction was applied to the dilute acid pretreated feedstock lines, suggesting that the inhibition is caused by water-soluble constituents.

The picture is quite different for the *Caldicellulosiruptor* sp. strains tested. Fermentation of dilute acid pretreated and hot water extracted biomass that was readily fermented by *C. thermocellum* caused significant reduction in fermentation yield for T1-2-TG and T1-3-TG substrates with both *Caldicellulosiruptor* sp. strains. In addition, there were only low levels of unconsumed sugar remaining in the broth at the end of fermentation, indicating that both fermentation and hydrolysis were negatively impacted for the two highly down-regulated COMT feedstock lines. Moreover, the apparent differential of fermentation inhibition between the three CBP microorganisms, measured by unconsumed carbohydrates or low product yields, was readily detected when a less severe hot water pretreatment was used to prepare the feedstock lines.

The apparent differential of inhibition between bacterial fermentations was particularly interesting because it was not seen in yeast-based SSF, and was an unexpected result. We hypothesize that the reduction in fermentation yield could be a biomass, microbe, or a biomass-microbe combined effect. A result that supports the hypothesis of a biomass effect contributing to the apparent inhibition is the significant reduction in yield of the *Caldicellulosiruptor* sp. strains’ fermentation of dilute acid pretreated, highly down-regulated COMT T1-2 and T1-3 lines, which is not present in the moderately down-regulated T1-12 transgenic line or the wild-type lines. Another possible reason for the apparent differential of inhibition is the various modes of interaction and hydrolysis employed by the hydrolytic system used by the microorganisms. As a result, they may release different or varying concentrations of inhibitory aromatic constituents, including mono-phenolic acids and sugar-aromatic conjugates. It is also not unreasonable to expect that the three microorganisms have different levels of tolerance for various inhibitory compounds.

We analyzed the fermentation broth and appropriate controls with GC-MS based metabolite profiling in an attempt to determine if mono-phenolic acids or other aromatic constituents were causing the observed inhibition. We showed temperature, media components, and fungal enzymes alone did not generate aromatic constituents or mono-phenolics, which are components of plant cell walls and known to inhibit bacterial fermentation [26,27]. The aromatic constituents, including mono-phenolic acids found in the fermentation broth for hot water versus dilute acid pretreatment are different. The variation in lignin derived constituents may be explained by the difference in pretreatment severity affecting the lignin structure and content [28].

In the case of hot water pretreatment, there was a mild biomass effect. Of specific interest was the increased relative abundance of aromatic constituents in the *Caldicellulosiruptor* sp. strains in comparison to *C. thermocellum*. This indicates that *C. thermocellum*’s hydrolytic system (cellulosome and free enzymes) might be producing a cleaner (less aromatic constituents) carbohydrate hydrolysate from the hot water pretreated switchgrass feedstocks than the *Caldicellulosiruptor* sp. strains. In contrast to the hot water pretreated feedstock results, dilute acid pretreated feedstocks did not show a notable difference in aromatic or mono-phenolic acid content between the different types of biomass or microorganisms. However, results showed that a tentatively identified compound, coumaroyl-benzaldehyde, was present in statistically different levels for both the biomass and microbe effect. The minimal biomass effect for either pretreatment was surprising, because our original hypothesis was based on the premise that the modification of the lignin pathway altered the lignin composition and content of the transgenic feedstock lines, and therefore, the concentration or composition of lignans generated and or released during pretreatment and bacterial hydrolysis and fermentation would appear quite different in comparison to the wild-type feedstock.

The differential of bacterial fermentation inhibition may, in part, be explained by the aromatic constituents in the fermentation broth. Additionally, it may also be explained by the microorganisms having varying degrees of tolerance to these compounds. In general, the reduction in recalcitrance drastically improved the susceptibility to bioconversion for yeast-based SSF and, after the inhibition was removed; high levels of fermentation products were produced by *C. thermocellum.* As a result, biomass sources with reduced recalcitrance resulting from lignin pathway modification are a valuable resource for producing economical biofuels, but the impact of the lignin modification on the three bacteria’s fermentation performance needs to be further studied to determine the cause of reduction in fermentation yield.

## Conclusions

In general, the reduction in recalcitrance drastically improved the susceptibility to hydrolysis and bioconversion for yeast-based SSF, and after removal of water soluble inhibitors, high levels of fermentation products were also produced by *C. thermocellum.* The *Caldicellulosiruptor* sp. strains yielded only lower levels of fermentation products under these conditions with the transgenic feedstocks. The differential between bacterial fermentation inhibition may, in part, be explained by different aromatic constituents in the fermentation broth. Additionally, it may also be explained by the microorganisms having varying degrees of tolerance to these compounds. Overall, it may be concluded that biomass sources with reduced recalcitrance resulting from lignin pathway modification are a valuable resource for producing economical biofuels. However, during characterization of new biomass sources, *in vitro* assays such as sugar release assays should be supplemented with *in vivo* fermentation tests which we have shown can detect inhibitory compounds present in the biomass hydrolysate. The exact source and nature of these inhibitory compounds impacting the fermentation performance of our CBP candidate microorganisms warrants further investigation.

## Materials and methods

### Growth and harvesting conditions for transgenic and control plant material

COMT down-regulated transgenic and control switchgrass (*Panicum virgatum*) lines were generated by the Samuel Roberts Noble Foundation. Down-regulation of the COMT gene and its effect on plant material composition, growth, and harvesting conditions were described previously in Fu et al. [12]. Briefly, independent T0 generation transgenic plants were produced and crossed with a wild-type plant to obtain progeny seeds designated as T1 lines. Both COMT RNAi positive (TG) and negative (null segregant) plants were identified from the progeny of each cross, and the null segregant plants were used as wild-type controls (WT) for analyses of the corresponding T1 transgenic plants. Transgenic lines T1-2-TG and T1-3-TG were heavily down-regulated in COMT activity, T1-12-TG was a moderately down-regulated line [12].

### Pretreatment

The biomass was milled in a Wiley mill using a 20 mesh screen. Dilute acid and hot water pretreatments were performed by soaking the biomass overnight in 0.5% H_2_SO_4_ for dilute acid pretreatment or Milli-Q water for hot water pretreatment at a ratio of 9 mL of acid or water per gram of dry biomass and centrifuged at 8000 rpm, 30 minutes, and 4°C in a Sorvall RC-5B refrigerated superspeed centrifuge (Dupont Instruments) [12]. The biomass was loaded at a ratio of 2.5 g dry biomass per tube into 10 cm x 1 cm hastelloy steel tubular pretreatment reactors (Industrial Alloys Plus, Inc.). The reactors were pre-heated in boiling water for 2 minutes and then transferred to a fluidized sand bath (Omega FSB1: Techne Co.) at the desired temperature, 180°C, for 7.5 minutes for DA pretreatment or for 25 minutes for HW pretreatment [12,29]. The reactors were cooled by quenching in an ice bath. The biomass was removed from the reactors and washed with 100 mL Milli-Q water per gram dry biomass. The biomass was stored at −20°C until fermentation.

In the case of the dilute acid pretreated switchgrass line, inhibition was observed in fermentations, and as a result, the biomass was subjected to a hot water extraction to remove inhibitory water soluble compounds. The biomass was soaked in Milli-Q water overnight in glass pressure tubes (Chemglass) and transferred to a fluidized sand bath at 80°C for ten minutes. The biomass was washed a second time with 100 mL of Milli-Q water per gram dry biomass and stored at −20°C until fermentation.

### Simultaneous saccharification and fermentation (SSF)

SSF of the pretreated control and transgenic switchgrass lines using *S. cerevisiae* D5A (ATCC 200062) and 15 FPU per gram cellulose of Spezyme CP and 25% volume ratio to Spezyme CP of Accellerase BG was performed according to previously described methods [12,30]. The enzymes were generously donated by Genencor International. Samples were not removed from the bottles during the fermentation. Instead, weight loss was used to monitor the progress of the fermentation as described previously by Mielenz et al. [28]. All fermentations were conducted in biological triplicate (SSF and CBP).

### Consolidated bioprocessing conversion

All CBP fermentations were cultivated with a uniform media and single batches of pretreated biomass minimizing the effects of nutrients, substrate accessibility, particle size, and pretreatment-generated compounds on fermentation performance.

The fermentation conditions were as follows for CBP microorganisms: *C. thermocellum* (ATCC 27405) temperature of 58°C, pH 7.00, and orbital shaking 125 rpm, *Caldicellulosiruptor obsidiansis* ATCC BAA-2073) temperature 75°C, pH 7.00, and orbital shaking 125 rpm, and *Caldicellulosiruptor bescii* (ATCC BAA-1888) temperature 75°C, pH 7.00, and orbital shaking 125 rpm. Fermentations were conducted in 125 mL anaerobic serum bottles with a 50 mL working volume. The media was composed of 0.336 g/L KCl, 0.25 g/L NH_4_Cl, 1.00 g/L MgSO_4_·7H_2_O, 1.70 g/L KH_2_PO_4_, 0.50 g/L C_7_H_14_NO_4_S, 0.15 g/L CaCl_2_·2H_2_O, 1.75 g/L Na_3_C_6_H_5_O_7_·2H_2_O, 0.6 g/L CH_4_N_2_O, 1.00 g/L L-cysteine HCl, 0.30 mg/L resazurin, and 2.0 mL of 1000x MTC minerals [31,32]. Bottles were loaded with 0.5 g of biomass on a dry basis and 47.25 mL of media and autoclaved for 30 minutes. The following components were added after sterilization 1.25 mL of 50x MTC vitamins [31,32], 0.25 mL of 10% wt/vol yeast extract, 0.25 mL of 1.0 M NaHCO_3_, and a 2.0% vol/vol inoculum. The inoculum was grown in 125 mL anaerobic serum bottles with 50 mL of the same media and a carbon source of 5.0 g/L Avicel (FMC BioPolymer) at 125 rpm and at the appropriate fermentation temperature. The growth profile of the inoculum was monitored by measuring total pellet protein using a BCA protein assay as described previously by Raman et al. [22]. The inoculum for the fermentations was in mid to late log phase of growth and had total pellet protein of approximately 175 μg/mL, 100 μg/mL, and 100 μg/mL for *C. thermocellum*, *C. bescii*, and *C. obsidiansis*, respectively (Additional file 3: Figure S1, S2, and S3).

As described previously for SSF, samples were not removed from the bottles during fermentation; instead weight loss was used to monitor the progress of the fermentation. Briefly, bottles were tarred and warmed for 1 hour to reach fermentation temperature and then vented for 20 seconds in an anaerobic chamber to determine the weight loss due to temperature increase. Following the initial venting, the bottles were vented at approximately 12 hours and 24 hours for 20 seconds and then at 24-hour or 48-hour intervals until the weight loss had stabilized.

### Analytical methods

Fermentation broth samples were analyzed for metabolites (acetic acid, lactic acid, and ethanol) and residual carbohydrates (cellobiose, glucose, xylose, arabinose) using a high performance liquid chromatography (HPLC) LaChrom Elite® system (Hitachi High Technologies America, Inc.) equipped with a refractive index detector (model L-2490). The products and carbohydrates were separated using an Aminex® HPX-87H column (Bio-Rad Laboratories, Inc.), at a flow rate 0.5 mL/min of 5.0 mM sulfuric acid and a column temperature of 60°C [12,22].

Raw biomass, biomass post pretreatment and washing, and fermentation residues were analyzed for carbohydrate composition using quantitative saccharification (quan sacch) assay ASTM E 1758–01 (ASTM 2003) and HPLC method NREL/TP 51–42623. Briefly, the samples were analyzed for carbohydrate composition using a high performance liquid chromatography (HPLC) LaChrom Elite® system (Hitachi High Technologies America, Inc.) equipped with a refractive index detector (model L-2490) and a UV–Vis detector (model L-2420). The carbohydrates (glucose, xylose, galactose, mannose, and arabinose) and pentose and hexose sugar degradation products (furfural and 5-hydroxy methyl furfural) were separated using an Aminex® HPX-87P column (Bio-Rad Laboratories, Inc.), at a 0.6 mL/min flow rate of water and a column temperature of 80°C [12]. The theoretical yield was calculated based on the initial fermentable carbohydrate loaded (glucose plus cellobiose for *C thermocellum*; all biomass sugars for *Caldicellulosiruptor* sp. strains) and under the assumption that all available carbohydrate was converted to fermentation products. The initial fermentable carbohydrate loaded was determined by the quantitative saccharification assay performed on the pretreated biomass before fermentation.

Metabolite analysis using gas chromatography–mass spectrometry (GC-MS) was conducted using 250 μL of supernatants of *C. thermocellum, C. bescii,* and *C. obsidiansis* cultures (grown on control or transgenic, T1-2, T1-3, or T1-12 switchgrass lines) and 15 μL of sorbitol (0.1001 g/100 mL aqueous internal standard) transferred by pipette to a vial, frozen at −20°C, and then lyophilized. The internal standard was added to correct for subsequent differences in derivatization efficiency and changes in sample volume during heating. Dried extracts were dissolved in 500 μL of silylation–grade acetonitrile followed by the addition of 500 μL N-methyl-N-trimethylsilyltrifluoroacetamide (MSTFA) with 1% trimethylchlorosilane (TMCS) (Thermo Scientific, Bellefonte, PA), and samples then heated for one hour at 70°C to generate trimethylsilyl (TMS) derivatives [33]. After two days, 1-μL aliquots were injected into an Agilent Technologies Inc. 5975C inert XL gas chromatograph-mass spectrometer, fitted with an Rtx®-5MS with Integra-Guard™ (5% diphenyl/95% dimethyl polysiloxane) 30 m x 250 μm x 0.25 μm film thickness capillary column. The standard quadrupole GC-MS was operated in the electron impact (70 eV) ionization mode, with 6 full-spectrum (50–650 Da) scans per second. Gas (helium) flow was 1.0 mL/min with the injection port configured in the splitless mode. The injection port, MS Source, and MS Quad temperatures were 250°C, 230°C, and 150°C, respectively. The initial oven temperature was held at 50°C for two minutes and was programmed to increase at 20°C per minute to 325°C and held for another 11 minutes, before cycling back to the initial conditions. A large user-created database (>1600 spectra) of mass spectral electron ionization (EI) fragmentation patterns of TMS-derivatized compounds, as well as the Wiley Registry 8th Edition combined with NIST 05 mass spectral database, were used to identify the metabolites of interest to be quantified. Peaks were reintegrated and reanalyzed using a key selected ion, characteristic m/z fragment, rather than the total ion chromatogram, to minimize integrating co-eluting metabolites. The extracted peaks of known metabolites were scaled back up to the total ion current using predetermined scaling factors. The scaling factor for the internal standard (sorbitol) was used for unidentified metabolites. Peaks were quantified by area integration and the concentrations were normalized to the quantity of the internal standard recovered, volume of sample processed, derivatized, and injected. Three replicate fermentation samples per switchgrass line per microbial strain were analyzed, and the metabolite data were averaged by strain on a given biomass type. Unidentified metabolites were denoted by their retention time as well as key m/z fragments. The P-value was calculated using the Student’s t-test and the comparison was between the means of sets of triplicates for constituents. A compound was highlighted if it concentration was statistically significantly different (P≤0.05) and had a greater than 2-fold difference. In addition, the calculation of the various ratios of constituents will occasionally yield division by 0 which is significant if it is a number divided by zero and not zero divided by zero.

## Abbreviations

TG: Transgenic; WT: Wild-type; COMT: Caffeic acid 3-*O*-methyltransferase; COB: *C. obsidiansis*; CT: *C. thermocellum*; CB: *C. bescii*; T1: Generation one; SSF: Simultaneous saccharification and fermentation; CBP: Consolidated bioprocessing; GC-MS: Gas chromatography–mass spectrometry; HW: Hot water pretreatment; DA: Dilute acid pretreatment; m/z: Mass to charge ratio; HPLC: High performance liquid chromatography; ATCC: American Type Culture Collection.

## Competing interests

The authors declare that they have no competing interests.

## Authors’ contributions

KLY helped plan the work, conducted the experiments and wrote the manuscript. MRJr assisted in medium development, data acquisition/analysis and edited the manuscript. TJT interpreted the GCMS data and edited the manuscript. NLE and MZM conducted the GCMS analysis, acquired and analyzed the data. CF constructed and grew, and provided the transgenic switchgrass. ZYW planned and directed the transgenic switchgrass production, and edited the manuscript. SDHB isolated the COB strain, assisted in medium development and its growth. JRM helped plan and conducted the experiments and edited the manuscript. All authors have read and approved the final manuscript.

## Supplementary Material

Additional file 1 **Table S1.** Performance comparison after fermentation of dilute acid pretreated T1-COMT switchgrass by *S. cerevisiae* -based SSF and CBP conversion by *C. thermocellum*, *C. bescii*, and *C. obsidiansis;* transgenic (TG); wild-type (WT) switchgrass. **Table S2.** Performance comparison after fermentation of hot water pretreated T1-COMT switchgrass by *S. cerevisiae* -based SSF and CBP conversion with *C. thermocellum*, *C. bescii*, and *C. obsidiansis*; transgenic (TG); wild-type (WT) switchgrass. Click here for file

Additional file 2 **Table S3.** Ratio of identified lignin constituents with a 2-fold comparable difference and p-value < 0.05 from transgenic (TG) versus wild-type (WT) T1-3 switchgrass fermentation after hot water pretreatment using specified microorganism (biomass effect). **Table S4.** Ratio of selected lignin constituents with a 2-fold comparable difference and p-value < 0.05 after fermentation of hot water pretreated T1-COMT switchgrass by *C. bescii* or *C. obsidiansis* versus *C. thermocellum* (microbe effect); transgenic (TG); wild-type (WT) switchgrass. **Table S5.** Ratio of selected lignin constituents with a 2-fold comparable difference and p-value < 0.05 after fermentation of hot water pretreated T1-COMT switchgrass comparing *Caldicellulosiruptor bescii* to *Caldicellulosiruptor obsidiansis* (microbe effect);. transgenic (TG); wild-type (WT) switchgrass. **Table S6.** Ratio of selected lignin constituents with a 2-fold comparable difference and p-value < 0.05 for dilute acid pretreated T1-COMT switchgrass lines after fermentation by specified microorganism (biomass effect); transgenic (TG); wild-type (WT) switchgrass. **Table S7.** Ratio of selected lignin constituents with a 2-fold comparable difference and p-value < 0.05 for *C. bescii* (CB) or *C. obsidiansis* (COB) versus *C. thermocellum* (CT) (microbe effect) after fermentation of specified dilute acid pretreated T1-COMT switchgrass lines; transgenic (TG); wild-type (WT) switchgrass. **Table S8.** Ratio of selected lignin constituents with a 2-fold comparable difference and p-value < 0.05 for *C. bescii* (CB) versus *C. obsidiansis* (COB) (microbe effect) after fermentation of specified dilute acid pretreated T1-COMT switchgrass lines; transgenic (TG); wild-type (WT) switchgrass. Click here for file

Additional file 3 **Figure S1.***C. thermocellum* growth profile on 5 g/L Avicel measured by total pellet protein using a BCA protein assay and the values are the average of three biological replicate fermentations. **Figure S2.***C. bescii* growth profile on 5 g/L Avicel measured by total pellet protein using a BCA protein assay and the values are the average of three biological replicate fermentations. **Figure S3.***C. obsidiansis* growth profile on 5 g/L Avicel measured by total pellet protein using a BCA protein assay and the values are the average of three biological replicate fermentations. Click here for file
