# Supplementary material for: Evaluation of the bioconversion of genetically modified switchgrass using simultaneous saccharification and fermentation and a consolidated bioprocessing approach
Source: Biotechnol Biofuels. 2012 Nov 12;5:81. doi: 10.1186/1754-6834-5-81 (PMC3503607; doi:10.1186/1754-6834-5-81)
Supplement: Additional file 1 — Table S1. Performance comparison after fermentation of dilute acid pretreated T1-COMT switchgrass by S. cerevisiae -based SSF and CBP conversion by C. thermocellum, C. bescii, and C. obsidiansis; transgenic (TG); wild-type (WT) switchgrass. Table S2. Performance comparison after fermentation of hot water pretreated T1-COMT switchgrass by S. cerevisiae -based SSF and CBP conversion with C. thermocellum, C. bescii, and C. obsidiansis; transgenic (TG); wild-type (WT) switchgrass. [file 1754-6834-5-81-S1.docx]

**Table S1**

| Microorganism (pretreatment and preparation) | Dilute Acid Pretreated Biomass | Total Products Yield (mg/g carbohydrate) | % of Theoretical Achieved | Product Ratio Acetic:Lactic:Ethanol | Fermentation Time (hours) |
| --- | --- | --- | --- | --- | --- |
| *S. cerevisiae*  *(DA and washed)* | T1-2-WT | 145.8 ± 1.7 | 29.2 | Not applicable  Ethanol only | 333 |
|  | T1-2-TG | 222.5 ± 1.3 | 44.5 |  |  |
|  | T1-3-WT | 168.3 ± 3.5 | 33.7 |  |  |
|  | T1-3-TG | 270.5 ± 3.9 | 54.1 |  |  |
|  | T1-12-WT | 187.3 ± 3.4 | 37.5 |  |  |
|  | T1-12-TG | 220.2 ± 2.4 | 44.0 |  |  |
| *C. thermocellum (DA and washed)* | T1-2-WT | 198.3 ± 7.7 | 39.7 | 13.9:1, 3.0:1 | 120 |
|  | T1-2-TG | 226.5 ± 8.5 | 45.3 | 24.0:1, 4.1:1 |  |
|  | T1-3-WT | 205.2 ± 11.9 | 41.0 | 13.5:1, 3.3:1 |  |
|  | T1-3-TG | 179.4 ± 11.1 | 35.9 | 6.1:1, 3.0:1 |  |
|  | T1-12-WT | 214.8 ± 5.5 | 43.0 | 16.9:1, 3.6:1 |  |
|  | T1-12-TG | 182.7 ± 7.1 | 36.5 | 8.9:1, 2.6:1 |  |
| *C. thermocellum (DA and hot water extracted)* | T1-2-WT | 235.5 ± 2.1 | 47.1 | 34.9:1, 2.3:1 | 119 |
|  | T1-2-TG | 293.3 ± 4.7 | 58.7 | 13.5:1, 3.1:1 |  |
|  | T1-3-WT | 257.1 ± 8.0 | 51.4 | 19.4:1, 2.1:1 |  |
|  | T1-3-TG | 313.5 ± 10.2 | 62.7 | 14.3:1, 2.6:1 |  |
|  | T1-12-WT | 254.8 ± 7.6 | 51.0 | 35.5:1, 2.4:1 |  |
|  | T1-12-TG | 297.7 ± 5.0 | 59.5 | 25.7:1, 2.4:1 |  |
| *C. bescii*  *(DA and hot water extracted)* | T1-2-WT | 286.9 ± 15.2 | 57.4 | 5.0:1, 52.4:1 | 326 |
|  | T1-2-TG | 20.7 ± 0.9 | 4.1 | 10.8, no ethanol |  |
|  | T1-3-WT | 301.4 ± 6.9 | 60.2 | 5.0,75.3 |  |
|  | T1-3-TG | 30.4 ± 6.9 | 6.1 | 7.7:1, no ethanol |  |
|  | T1-12-WT | 268.3 ± 13.2 | 53.7 | 5.5:1, 63.3:1 |  |
|  | T1-12-TG | 270.6 ± 14.6 | 54.1 | 4.9:1, 639.4:1 |  |
| *C. obsidiansis*  *(DA and hot water extracted)* | T1-2-WT | 234.4 ± 6.4 | 46.9 | 72.4:1, 69.9:1 | 317 |
|  | T1-2-TG | 12.1 ± 0.3 | 2.4 | No Lactic or Ethanol |  |
|  | T1-3-WT | 252.9 ± 5.2 | 50.6 | 52.6:1, 75.8:1 |  |
|  | T1-3-TG | 19.4 ± 6.0 | 3.9 | No Lactic or Ethanol |  |
|  | T1-12-WT | 247.3 ± 5.8 | 49.5 | 43.7:1, 92.0:1 |  |
|  | T1-12-TG | 240.2 ± 17.2 | 48.0 | 54.4:1, 65.9:1 |  |

**Table S2**

| Microorganism | Hot Water Pretreated Biomass | Total Products Yield  (mg/g carbohydrate) | % of Theoretical Achieved | Product Ratio Acetic:Lactic:Ethanol | Fermentation Time (hours) |
| --- | --- | --- | --- | --- | --- |
| *S. cerevisiae* | T1-2-WT | 131.0 ± 8.2 | 26.2 | Not applicable  Ethanol only | 333 |
|  | T1-2-TG | 161.1 ± 2.6 | 32.2 |  |  |
|  | T1-3-WT | 125.3 ± 5.6 | 25.1 |  |  |
|  | T1-3-TG | 192.5 ± 8.5 | 38.5 |  |  |
|  | T1-12-WT | 128.2 ± 6.5 | 25.6 |  |  |
|  | T1-12-TG | 157.0 ± 6.3 | 31.4 |  |  |
| *C. thermocellum* | T1-3-WT | 210.9 ± 4.6 | 42.2 | 21.0:1, 2.80:1 | 206 |
|  | T1-3-TG | 233.0 ± 4.0 | 46.6 | 20.0:1, 2.90:1 |  |
| *C. bescii* | T1-3-WT | 62.9 ± 21.2 | 12.6 | 3.25:1, No Ethanol Detected | 667 |
|  | T1-3-TG | 46.5 ± 1.1 | 9.3 | 3.00:1, No Ethanol Detected |  |
| *C. obsidiansis* | T1-3-WT | 235.1 ± 10.1 | 47.0 | 16.5:1, No Ethanol Detected | 667 |
|  | T1-3-TG | 243.9 ± 8.9 | 48.8 | 13.6:1, No Ethanol Detected |  |
