# Supplementary material for: Evaluation of the bioconversion of genetically modified switchgrass using simultaneous saccharification and fermentation and a consolidated bioprocessing approach
Source: Biotechnol Biofuels. 2012 Nov 12;5:81. doi: 10.1186/1754-6834-5-81 (PMC3503607; doi:10.1186/1754-6834-5-81)
Supplement: Additional file 2 — Table S3. Ratio of identified lignin constituents with a 2-fold comparable difference and p-value < 0.05 from transgenic (TG) versus wild-type (WT) T1-3 switchgrass fermentation after hot water pretreatment using specified microorganism (biomass effect). Table S4. Ratio of selected lignin constituents with a 2-fold comparable difference and p-value < 0.05 after fermentation of hot water pretreated T1-COMT switchgrass by C. bescii or C. obsidiansis versus C. thermocellum (microbe effect); transgenic (TG); wild-type (WT) switchgrass. Table S5. Ratio of selected lignin constituents with a 2-fold comparable difference and p-value < 0.05 after fermentation of hot water pretreated T1-COMT switchgrass comparing Caldicellulosiruptor bescii to Caldicellulosiruptor obsidiansis (microbe effect);. transgenic (TG); wild-type (WT) switchgrass. Table S6. Ratio of selected lignin constituents with a 2-fold comparable difference and p-value < 0.05 for dilute acid pretreated T1-COMT switchgrass lines after fermentation by specified microorganism (biomass effect); transgenic (TG); wild-type (WT) switchgrass. Table S7. Ratio of selected lignin constituents with a 2-fold comparable difference and p-value < 0.05 for C. bescii (CB) or C. obsidiansis (COB) versus C. thermocellum (CT) (microbe effect) after fermentation of specified dilute acid pretreated T1-COMT switchgrass lines; transgenic (TG); wild-type (WT) switchgrass. Table S8. Ratio of selected lignin constituents with a 2-fold comparable difference and p-value < 0.05 for C. bescii (CB) versus C. obsidiansis (COB) (microbe effect) after fermentation of specified dilute acid pretreated T1-COMT switchgrass lines; transgenic (TG); wild-type (WT) switchgrass. [file 1754-6834-5-81-S2.docx]

**Table S3**

|  | *C. bescii* TG/WT | | *C. obsidiansis* TG/WT | | *C. thermocellum* TG/WT | |
| --- | --- | --- | --- | --- | --- | --- |
| Aromatic constituent (µg/mL) sorbitol equivalents | Ratio | P-value | Ratio | P-value | Ratio | P-value |
| Arabitol | 0.91 | 0.84 | 0.75 | 0.12 | 0.71 | 0.00038 |
| arabitol-phenolic conjugate | 0.091 | 0.29 | 2.01 | 0.071 | 0.72 | 0.00024 |
| p-coumaric acid | 0.93 | 0.67 | 0.91 | 0.69 | 0.56 | 0.031 |
| C5-sinapyl conjugate | 1.68 | 0.036 | 2.35 | 0.0087 | 1.81 | 0.20 |
| sinapyl alcohol | 0.53 | 0.054 | 0.69 | 0.0032 | 0.8225 | 0.0050 |
| 5-hydroxyconiferyl alcohol | 0.70 | 0.01 | 1.14 | 0.08 | 0.69 | 0.54 |
| xylotriose -sinapyl alcohol conjugate | 0.86 | 0.92 | NA | NA | 0.73 | 0.0050 |
| coniferyl alcohol | 0.87 | 0.075 | 1.03 | 0.46 | 0.82 | 0.025 |

**Table S4**

|  | *C. bescii* TG / *C. thermocellum* TG | | *C. obsidiansis* TG / *C. thermocellum* TG | | *C. bescii* WT / *C. thermocellum* WT | | *C. obsidiansis* WT / *C. thermocellum* WT | |
| --- | --- | --- | --- | --- | --- | --- | --- | --- |
| Aromatic constituent (µg/mL) sorbitol equivalents | Ratio | P-value | Ratio | P-value | Ratio | P-value | Ratio | P-value |
| Arabitol | 0.11 | 0.0000026 | 1.27 | 0.28 | 0.086 | 0.000029 | 1.19 | 0.0034 |
| arabitol-phenolic conjugate | 0.088 | 0.0000040 | 2.29 | 0.052 | 0.69 | 0.58 | 0.82 | 0.00022 |
| p-coumaric acid | 0.43 | 0.0026 | 0.31 | 0.0026 | 0.26 | 0.0050 | 0.19 | 0.0033 |
| C5-sinapyl conjugate | 67.17 | 0.000037 | 8.58 | 0.0013 | 72.34 | 0.0081 | 6.61 | 0.0036 |
| sinapyl alcohol | 0.73 | 0.0012 | 0.88 | 0.17 | 1.15 | 0.51 | 1.06 | 0.064 |
| 5-hydroxyconiferyl alcohol | 17.01 | 0.00038 | 19.66 | 0.000024 | 16.66 | 0.000023 | 11.92 | 0.000055 |
| xylotriose -sinapyl alcohol conjugate | 0.0046 | 0.00006 | NA | NA | 0.0039 | 0.0000036 | NA | NA |
| coniferyl alcohol | 3.68 | 0.000066 | 2.49 | 0.000074 | 3.44 | 0.000064 | 1.97 | 0.000077 |

**Table S5**

|  | *C. bescii* TG / *C. obsidiansis* TG | | *C. bescii* WT / *C. obsidiansis* WT | | *C. obsidianisis* TG / *C. bescii* TG | | *C. obsidianisis* WT / *C. bescii* WT | |
| --- | --- | --- | --- | --- | --- | --- | --- | --- |
| Aromatic constituent (µg/mL) sorbitol equivalents | Ratio | P-value | Ratio | P-value | Ratio | P-value | Ratio | P-value |
| Arabitol | 0.087 | 0.005 | 0.072 | 0.000 | 11.505 | 0.005 | 13.862 | 0.000 |
| arabitol-phenolic conjugate | 0.038 | 0.009 | 0.848 | 0.819 | 26.106 | 0.009 | 1.180 | 0.819 |
| p-coumaric acid | 1.393 | 0.193 | 1.358 | 0.130 | 0.718 | 0.193 | 0.736 | 0.130 |
| C5-sinapyl conjugate | 7.8284 | 0.0001 | 10.9519 | 0.0108 | 0.1277 | 0.0001 | 0.0913 | 0.0108 |
| sinapyl alcohol | 0.8310 | 0.0802 | 1.0816 | 0.6897 | 1.2033 | 0.0802 | 0.9245 | 0.6897 |
| 5-hydroxyconiferyl alcohol | 0.8651 | 0.1528 | 1.3978 | 0.0043 | 1.1559 | 0.1528 | 0.7154 | 0.0043 |
| xylotriose-sinapyl alcohol conjugate | NA | NA | NA | NA | NA | NA | NA | NA |
| coniferyl alcohol | 1.480 | 0.003 | 1.748 | 0.000 | 0.676 | 0.003 | 0.572 | 0.000 |

**Table S6**

| Ratio of TG/WT | xylitol | arabitol | *cis*-p-coumaric acid | *trans*-p-coumaric acid | coumaroyl-benzaldehyde | ferulic acid | caffeic acid | sinapyl alcohol |
| --- | --- | --- | --- | --- | --- | --- | --- | --- |
| *C. bescii* T1-2 | 0.46 | 0.17 | 0.66 | 0.84 | 3.00 | 1.26 | 1.90 | 0.70 |
| P Value | 0.02 | 0.01 | 0.03 | 0.49 | 0.00 | 0.37 | 0.01 | 0.29 |
| *C. bescii* T1-3 | 0.32 | 0.17 | 0.68 | 0.63 | 2.54 | 0.84 | 1.40 | 0.55 |
| P-Value | 0.00 | 0.00 | 0.11 | 0.04 | 0.01 | 0.31 | 0.03 | 0.01 |
| *C. bescii* T1-12 | 0.91 | 0.70 | 0.77 | 0.64 | 0.80 | 0.71 | 1.24 | 0.61 |
| P-value | 0.33 | 0.05 | 0.03 | 0.01 | 0.03 | 0.02 | 0.02 | 0.00 |
| *C. obsidiansis* T1-2 | 0.07 | 0.13 | 1.13 | 1.11 | 3.48 | 1.26 | 1.71 | 1.02 |
| P Value | 0.00 | 0.00 | 0.52 | 0.12 | 0.00 | 0.01 | 0.01 | 0.86 |
| *C. obsidiansis* T1-3 | 0.13 | 0.20 | 1.46 | 1.17 | 3.71 | 1.28 | 1.55 | 1.51 |
| P-Value | 0.00 | 0.00 | 0.01 | 0.00 | 0.00 | 0.00 | 0.00 | 0.04 |
| *C. obsidiansis* T1-12 | 0.82 | 1.33 | 1.16 | 0.91 | 1.47 | 0.83 | 1.10 | 1.01 |
| P-value | 0.15 | 0.05 | 0.58 | 0.09 | 0.03 | 0.03 | 0.31 | 0.62 |
| *C. thermocellum* T1-2 | 0.40 | 0.42 | 0.67 | 0.80 | 0.00 | 0.80 | 1.22 | 0.91 |
| P-value | 0.00 | 0.00 | 0.05 | 0.00 | 0.37 | 0.00 | 0.07 | 0.61 |
| *C. thermocellum* T1-3 | 0.64 | 0.63 | 0.96 | 1.18 | NA | 1.11 | 1.18 | 1.08 |
| P-value | 0.17 | 0.15 | 0.89 | 0.59 | 0.00 | 0.73 | 0.61 | 0.87 |
| *C. thermocellum* T1-12 | 0.74 | 0.70 | 1.00 | 1.01 | NA | 0.87 | 1.07 | 1.02 |
| P-value | 0.03 | 0.01 | 0.98 | 0.94 | 0.00 | 0.28 | 0.65 | 0.90 |

**Table S7**

|  | xylitol | arabitol | *cis*-p-coumaric acid | *trans*-p-coumaric acid | coumaroyl-benzaldehyde | ferulic acid | caffeic acid | sinapyl alcohol |
| --- | --- | --- | --- | --- | --- | --- | --- | --- |
| CB/CT T1-2-WT | 0.25 | 0.41 | 1.21 | 0.22 | 12.42 | 0.18 | 0.33 | 1.54 |
| P-value | 0.00 | 0.00 | 0.24 | 0.00 | 0.03 | 0.00 | 0.00 | 0.26 |
| CB/CT T1-2-TG | 0.29 | 0.16 | 1.19 | 0.23 | NA | 0.29 | 0.52 | 1.18 |
| P-value | 0.00 | 0.00 | 0.15 | 0.00 | 0.00 | 0.00 | 0.00 | 0.00 |
| CB/CT T1-3-WT | 0.35 | 0.44 | 1.31 | 0.29 | NA | 0.26 | 0.31 | 2.45 |
| P-value | 0.04 | 0.06 | 0.35 | 0.06 | 0.00 | 0.06 | 0.09 | 0.03 |
| CB/CT T1-3-TG | 0.18 | 0.12 | 0.94 | 0.15 | 5.74 | 0.19 | 0.37 | 1.26 |
| P-value | 0.00 | 0.00 | 0.74 | 0.00 | 0.00 | 0.00 | 0.00 | 0.35 |
| CB/CT T1-12-WT | 0.22 | 0.50 | 0.96 | 0.23 | NA | 0.20 | 0.32 | 2.41 |
| P-value | 0.00 | 0.00 | 0.56 | 0.00 | 0.00 | 0.00 | 0.01 | 0.00 |
| CB/CT T1-12-TG | 0.27 | 0.50 | 0.74 | 0.14 | 2.93 | 0.16 | 0.37 | 1.44 |
| P-value | 0.00 | 0.00 | 0.03 | 0.00 | 0.00 | 0.00 | 0.00 | 0.03 |
| COB/CT T1-2-WT | 0.64 | 0.43 | 0.74 | 0.15 | 10.64 | 0.17 | 0.36 | 0.97 |
| P-value | 0.00 | 0.00 | 0.14 | 0.00 | 0.00 | 0.00 | 0.00 | 0.85 |
| COB/CT T1-2-TG | 0.10 | 0.13 | 1.24 | 0.22 | NA | 0.27 | 0.51 | 1.08 |
| P-value | 0.00 | 0.00 | 0.21 | 0.00 | 0.00 | 0.00 | 0.00 | 0.48 |
| COB/CT T1-3-WT | 0.71 | 0.46 | 0.64 | 0.17 | NA | 0.18 | 0.29 | 1.18 |
| P-value | 0.26 | 0.06 | 0.26 | 0.04 | 0.00 | 0.04 | 0.09 | 0.70 |
| COB/CT T1-3-TG | 0.14 | 0.15 | 0.97 | 0.17 | 5.88 | 0.20 | 0.38 | 1.65 |
| P-value | 0.00 | 0.00 | 0.56 | 0.00 | 0.00 | 0.00 | 0.00 | 0.04 |
| COB/CT T1-12-WT | 0.48 | 0.45 | 0.53 | 0.15 | NA | 0.16 | 0.36 | 1.18 |
| P-value | 0.00 | 0.00 | 0.01 | 0.00 | 0.00 | 0.00 | 0.01 | 0.23 |
| COB/CT T1-12-TG | 0.53 | 0.86 | 0.62 | 0.14 | 4.31 | 0.16 | 0.37 | 1.17 |
| P-value | 0.01 | 0.19 | 0.04 | 0.00 | 0.00 | 0.00 | 0.00 | 0.21 |

**Table S8**

|  | xylitol | arabitol | *cis*-p-coumaric acid | *trans*-p-coumaric acid | coumaroyl-benzaldehyde | ferulic acid | caffeic acid | sinapyl alcohol |
| --- | --- | --- | --- | --- | --- | --- | --- | --- |
| CB/COB T1-2-WT | 0.39 | 0.95 | 1.65 | 1.42 | 1.17 | 1.07 | 0.91 | 1.59 |
| P-value | 0.00 | 0.78 | 0.03 | 0.23 | 0.62 | 0.82 | 0.65 | 0.21 |
| CB/COB T1-2-TG | 2.77 | 1.28 | 0.96 | 1.08 | 1.01 | 1.06 | 1.01 | 1.09 |
| P-value | 0.00 | 0.02 | 0.77 | 0.27 | 0.94 | 0.37 | 0.91 | 0.36 |
| CB/COB T1-3-WT | 0.50 | 0.95 | 2.07 | 1.68 | 1.42 | 1.46 | 1.05 | 2.07 |
| P-value | 0.00 | 0.69 | 0.00 | 0.02 | 0.16 | 0.04 | 0.54 | 0.00 |
| CB/COB T1-3-TG | 1.26 | 0.80 | 0.97 | 0.90 | 0.98 | 0.95 | 0.95 | 0.76 |
| P-value | 0.14 | 0.30 | 0.88 | 0.35 | 0.84 | 0.67 | 0.58 | 0.19 |
| CB/COB T1-12-WT | 0.45 | 1.11 | 1.79 | 1.50 | 1.24 | 1.22 | 0.89 | 2.04 |
| P-value | 0.00 | 0.17 | 0.02 | 0.00 | 0.05 | 0.01 | 0.07 | 0.00 |
| CB/COB T1-12-TG | 0.50 | 0.58 | 1.19 | 1.06 | 0.68 | 1.04 | 1.00 | 1.23 |
| P-value | 0.01 | 0.02 | 0.35 | 0.68 | 0.03 | 0.77 | 1.00 | 0.01 |
