# Supplementary material for: Evaluation of the bioconversion of genetically modified switchgrass using simultaneous saccharification and fermentation and a consolidated bioprocessing approach
Source: Biotechnol Biofuels. 2012 Nov 12;5:81. doi: 10.1186/1754-6834-5-81 (PMC3503607; doi:10.1186/1754-6834-5-81)
Supplement: Additional file 3 — Figure S1.C. thermocellum growth profile on 5 g/L Avicel measured by total pellet protein using a BCA protein assay and the values are the average of three biological replicate fermentations. Figure S2.C. bescii growth profile on 5 g/L Avicel measured by total pellet protein using a BCA protein assay and the values are the average of three biological replicate fermentations. Figure S3.C. obsidiansis growth profile on 5 g/L Avicel measured by total pellet protein using a BCA protein assay and the values are the average of three biological replicate fermentations. [file 1754-6834-5-81-S3.docx]

Figure S1

Figure S2

Figure S3
